# Supplementary figures and images for: ARID1A regulates R-loop associated DNA replication stress
Source: PLoS Genet. 2021 Apr 7;17(4):e1009238. doi: 10.1371/journal.pgen.1009238 (PMC8055027; doi:10.1371/journal.pgen.1009238)

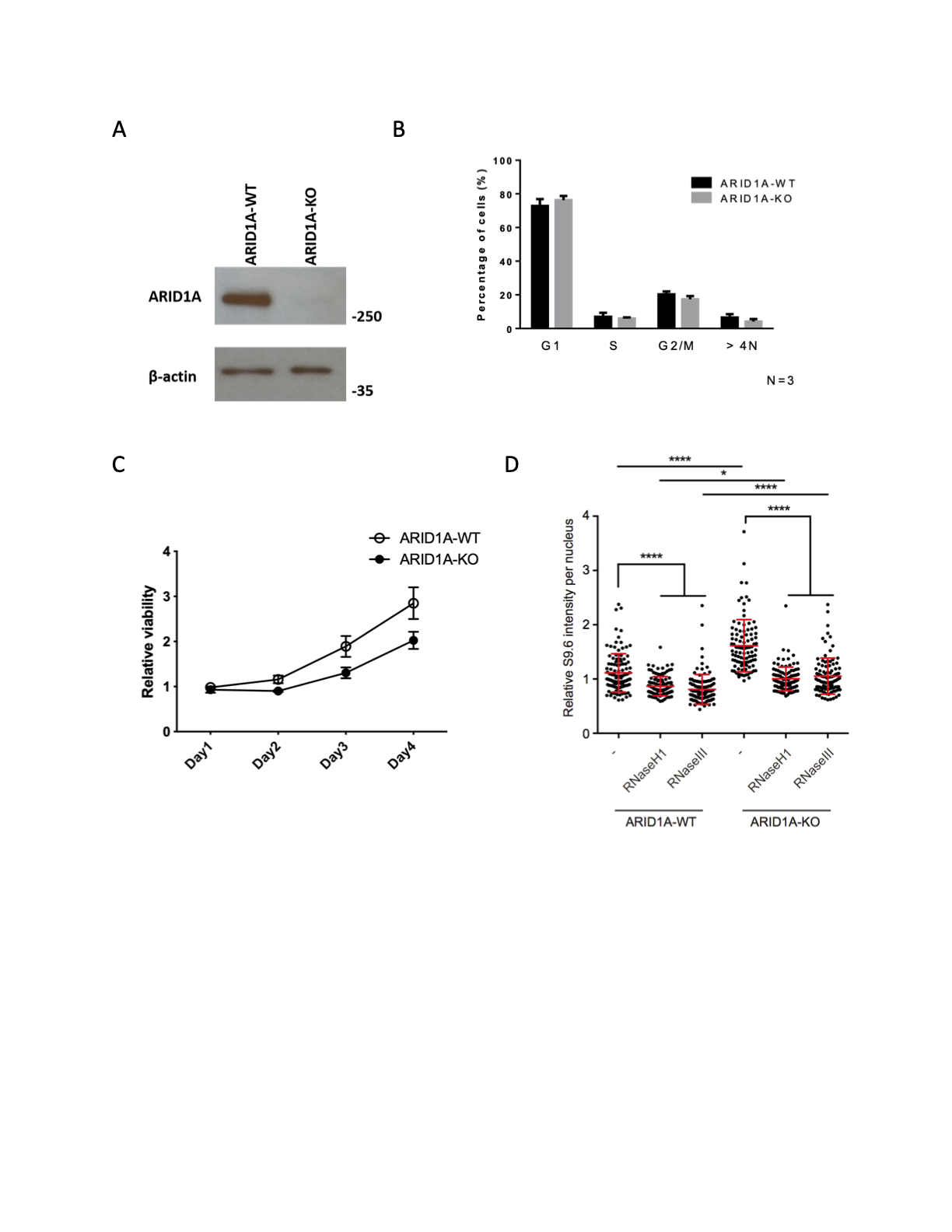

Supplement: S1 Fig — Immunoblots of ARID1A and β-actin proteins in RMG1 wild type (ARID1A-WT) or ARID1A crispr knockout RMG1 (ARID1A-KO) cells. β-Actin is a loading control. Representative blot of N = 3 biological repeats. (B) Cell cycle distribution of RMG1 and ARID1A-KO cells based on flow cytometry of DNA content. No significant differences were observed. (C) Relative viability over four days as measured by crystal violet staining of cell number shows slowed growth of the ARID1A-KO cells. (D) Effect of RNaseH1 and RNaseIII treatment on S9.6 staining in ARID1A-WT and ARID1A-KO cells. Nucleolar S9.6 staining was subtracted from the total nuclear intensity. Both treatments reduce overall signal but ARID1A-KO have a significant RNaseIII resistant staining increase consistent with DNA:RNA hybrid accumulation. (TIF) [file pgen.1009238.s001.tif]

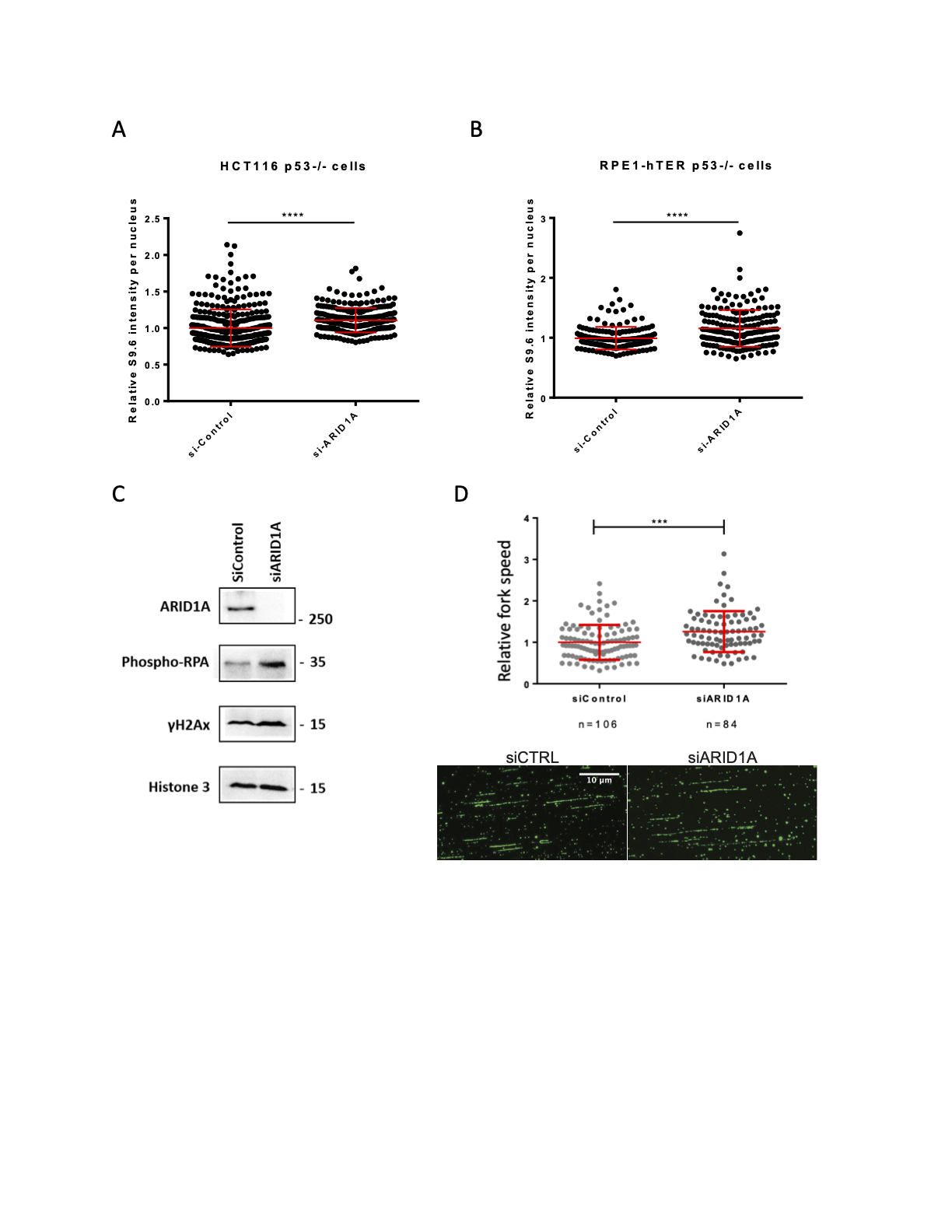

Supplement: S2 Fig — (A) ARID1A loss induced more R-loops in both HCT116 and (B) RPE1-hTERT p53 negative cells. HCT116 p53-/- cells (A) or RPE1-hTERT p53 -/- cells (B) were transfected with si-Control or ARID1A siRNA for two days then were fixed and stained with s9.6 and DAPI. Quantification of S9.6 staining per nucleus was measured by ImageJ. (A = siControl 330 nuclei, siARID1A 265 nuclei; B = siControl 153 nuclei, siARID1A 181 nuclei). Composite of N = 3; ****p<0.0001 by t test; mean ± SD. (C) Total protein extracts shows that ARID1A loss in HCT116 p53 negative cells has more RPA2-ser33P and γH2Ax. (D) ARID1A loss in HCT116 p53 negative cells induced higher replication fork speed measured by Quantifying DNA fiber. N = 2; ***p<0.001 by unpaired T-test; mean ± SD. (TIF) [file pgen.1009238.s002.tif]

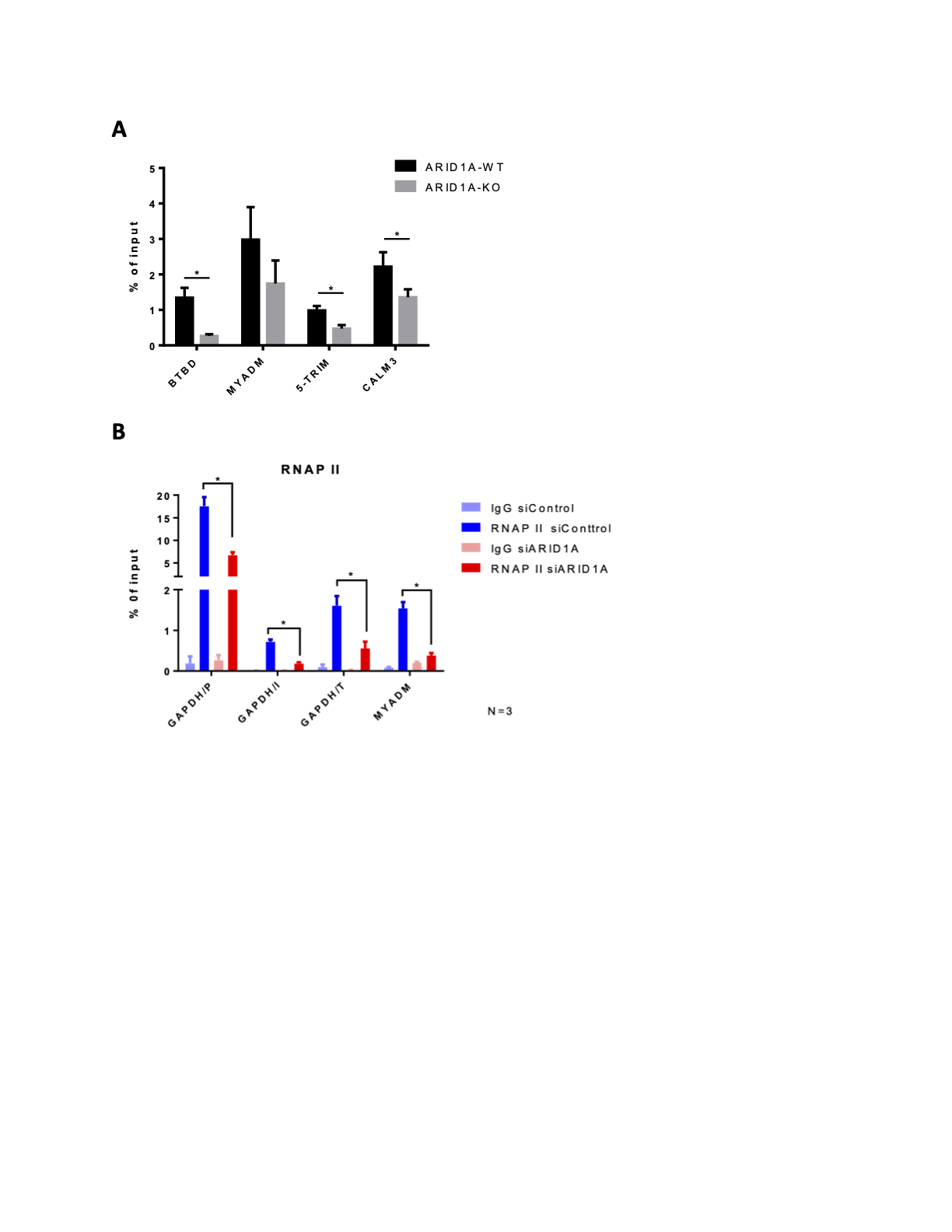

Supplement: S3 Fig — (A) ChIP-qPCR probing for RNAP II in WT and ARID1A-KO RMG1 cells showing loss of RNAP II binding at both R-loop prone loci (BTBD and MYADM) and DRIP-negative sites (5’-TRIM and CALM3). N = 3; *p<0.05 by t test; mean ± SEM. (B) ChIP-qPCR for RNAPII in HCT116 p53-/- control siRNA treated cells (blue) or ARID1A siRNA depleted cells (red). Both ARID1A positive sites (GAPDH/P and MYADM) and ARID1A negative sites (GAPDH/I and GAPDH/T) show lower RNAP II binding. (TIF) [file pgen.1009238.s003.tif]

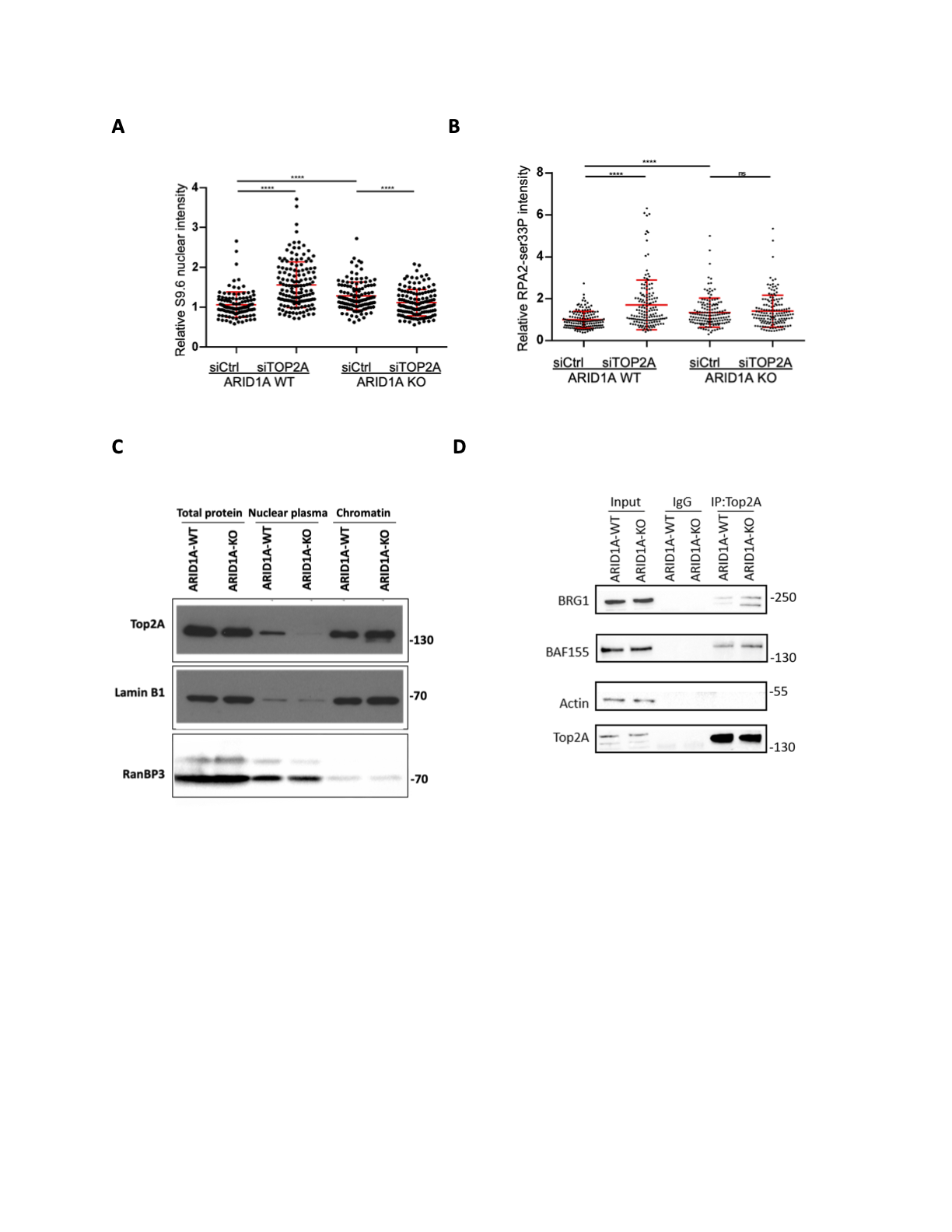

Supplement: S4 Fig — (A) siRNA depletion of TOP2A increases S9.6 nuclear staining intensity in RMG1 WT but not ARID1A-KO cells. Shown is a composite of three replicates. N = 3; p <0.0001, ANOVA with Tukey’s post-hoc test. (B) siRNA depletion of TOP2A increases RPA2-ser33P staining intensity. Shown is a composite of three replicates. N = 3; p <0.0001, ANOVA with Tukey’s post-hoc test. ns = not significant. (C) Chromatin fractionation of ARID1A-WT or ARID1A-KO RMG1 cells shows that TOP2A protein levels are not affected by ARID1A loss, and that TOP2A is still strongly associated with chromatin. LaminB1 is included as a control associated with insoluble nuclear material, including chromatin. RanBP3 is included as a control for soluble nuclear material. (D) Immunoprecipitation of TOP2A in ARID1A-WT and KO RMG1 cell lines. Actin is included as an input loading control, TOP2A is shown to confirm robust pulldown. Brg1 and BAF155 co-precipitated with TOP2A relative to an IgG control regardless of ARID1A-WT or KO status. For C and D, molecular weight marker positions are shown (right). (TIF) [file pgen.1009238.s004.tif]

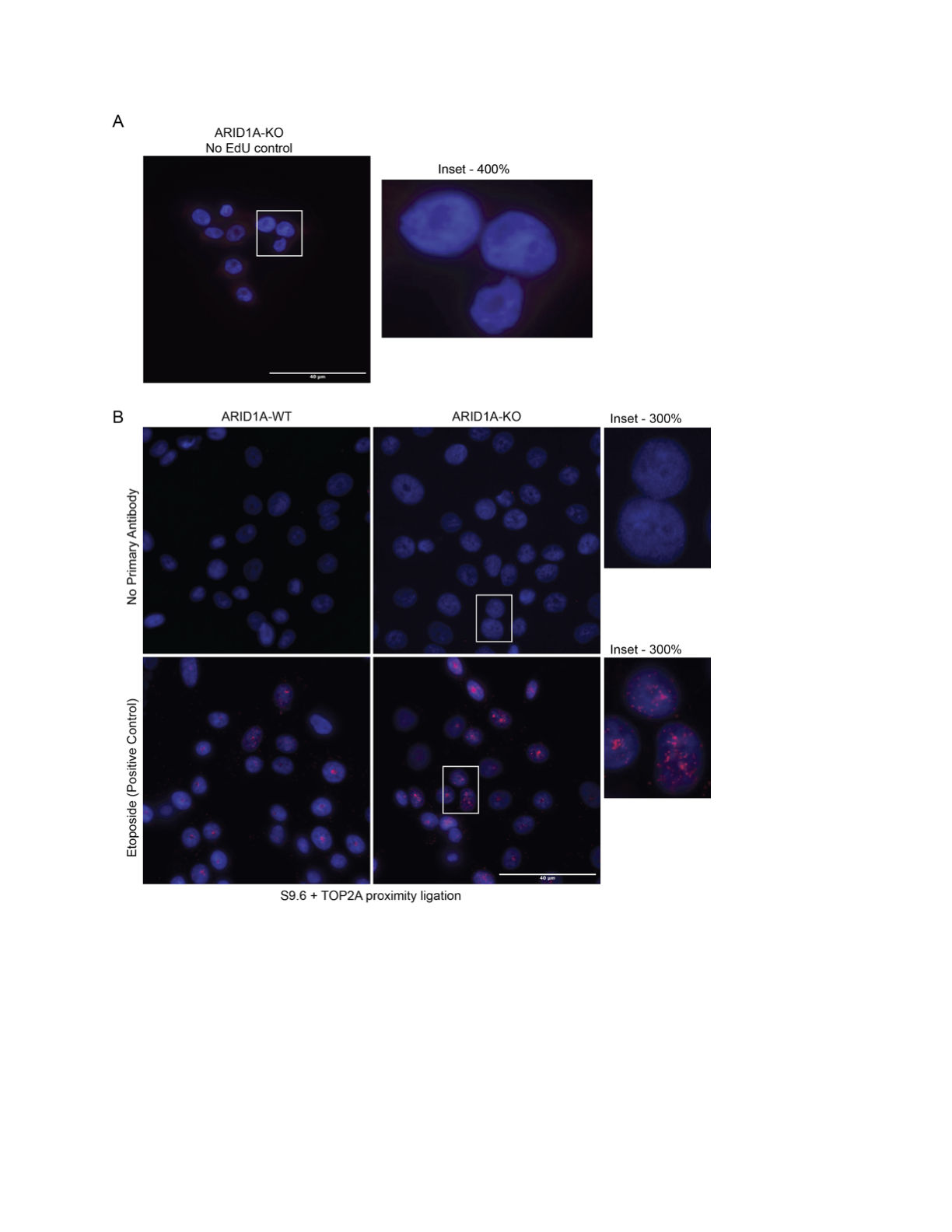

Supplement: S5 Fig — (A) SIRF control images of ARID1A-KO cells that followed the MRE11 SIRF procedure without an EdU treatment pulse before fixation. No nuclear staining or foci are ever seen. A zoomed image of DAPI stained nuclei are shown on the right. (B) Negative control images lacking primary antibodies (top), or with etoposide treatment (as in Fig 4) are shown for the S9.6-TOP2A antibody PLA reactions. Zoomed inset images (right) show no background signal without antibody addition, but very strong focal staining after Etoposide treatment. (TIF) [file pgen.1009238.s005.tif]

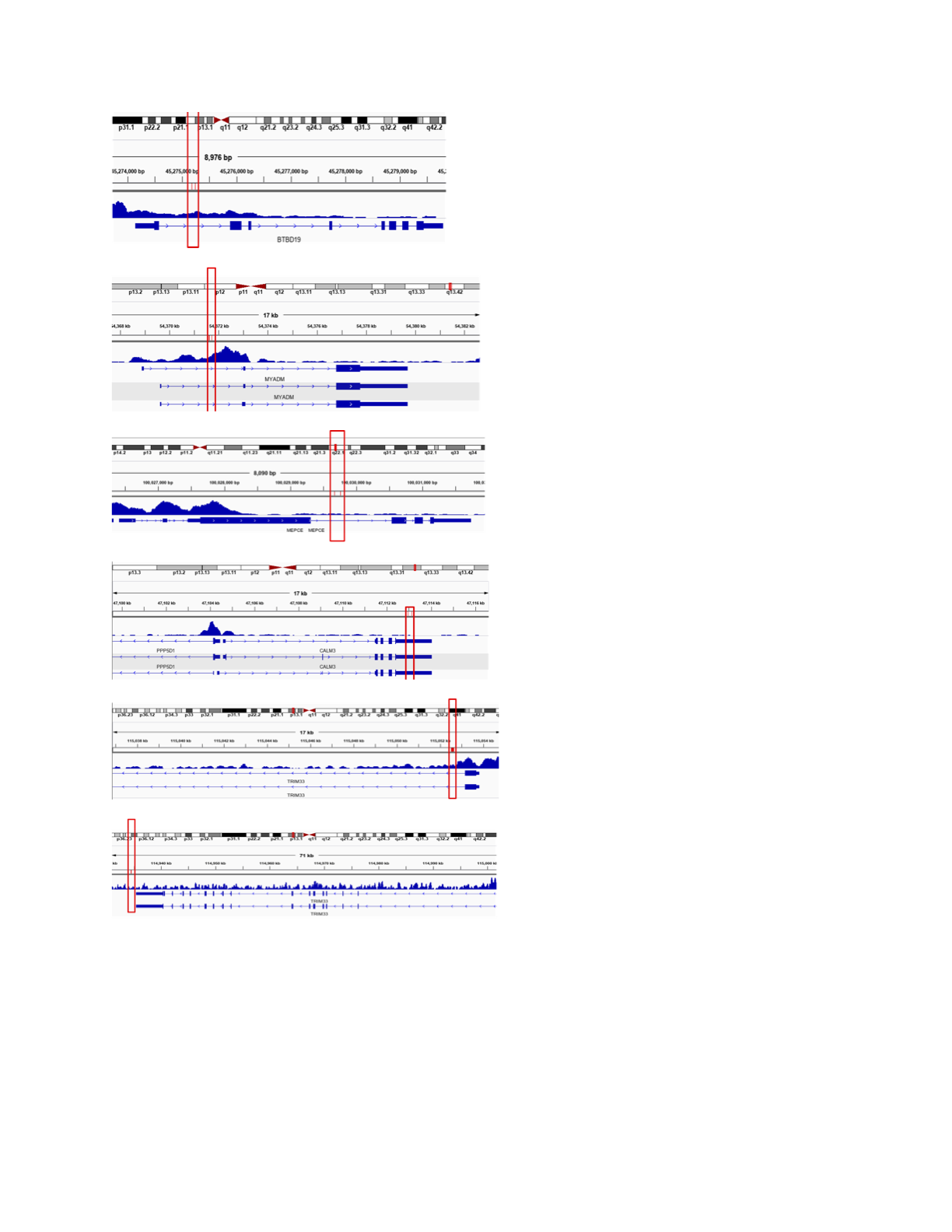

Supplement: S6 Fig — From top to bottom BTBD19; MYADM; MEPCE; CALM3; 5’ TRIM33; 3’ TRIM33. Red boxes indicate the PCR amplicon. The blue track represents ARID1A ChIP-seq signal from reference 47 (GEO: GSM3392689). (TIF) [file pgen.1009238.s006.tif]
